# RB1CC1 Activates RB1 Pathway and Inhibits Proliferation and Cologenic Survival in Human Cancer

**DOI:** 10.1371/journal.pone.0011404

**Published:** 2010-06-30

**Authors:** Tokuhiro Chano, Kaichiro Ikebuchi, Yasuko Ochi, Hitosuke Tameno, Yasuhiko Tomita, Yufen Jin, Hideo Inaji, Makoto Ishitobi, Koji Teramoto, Ichiro Nishimura, Kahori Minami, Hirokazu Inoue, Takahiro Isono, Masao Saitoh, Taketoshi Shimada, Yasuo Hisa, Hidetoshi Okabe

**Affiliations:** 1 Department of Clinical Laboratory Medicine, Shiga University of Medical Science, Otsu, Japan; 2 Department of Otolaryngology-Head and Neck Surgery, Kyoto Prefectural University of Medicine, Kyoto, Japan; 3 Department of Pathology, Osaka Medical Center for Cancer and Cardiovascular Diseases, Osaka, Japan; 4 Department of Breast and Endocrine Surgery, Osaka Medical Center for Cancer and Cardiovascular Diseases, Osaka, Japan; 5 Department of Thoracic Surgery, Shiga University of Medical Science, Otsu, Japan; 6 Department of Microbiology, Shiga University of Medical Science, Otsu, Japan; 7 Central Research Laboratory, Shiga University of Medical Science, Otsu, Japan; 8 Frontier Research on Molecular Destruction and Reconstruction of Tooth and Bone, Tokyo Medical and Dental University, Tokyo, Japan; Bauer Research Foundation, United States of America

## Abstract

*RB1*-inducible coiled-coil 1 (RB1CC1, also known as FIP200) plays a role in the enhancement of the RB1 pathway through the direct binding to a GC-rich region 201bp upstream (from the initiation ATG) of the *RB1* promoter. Here, we identified hSNF5 and p53 as the binding partners of RB1CC1 by immunoprecipitation and immunofluorescence assays. Interaction between these molecules and the RB1 pathway was analyzed by the assays of chromatin immunoprecipitation, luciferase-reporter, reverse transcription-polymerase chain reaction and immunoblot. The tumor growth suppression by RB1CC1 was evaluated by flow cytometry or by a cell growth assay. The nuclear RB1CC1 complex involving hSNF5 and/or p53 activated transcription of *RB1*, *p16* and *p21*, and suppressed tumor cell growth. Furthermore, nuclear RB1CC1 expression significantly correlated with those of RB1 and p16 in breast cancer tissue *in vivo*, and the Ki-67 proliferation index was dependent on p53 as well as RB1CC1. The present study indicates that RB1CC1 together with hSNF5 and/or p53 enhances the RB1 pathway through transcriptional activation of *RB1*, *p16* and *p21*. Evaluation of RB1CC1 expression combined with RB1 and p53 status is expected to provide useful information in clinical practice and future therapeutic strategies in breast cancer.

## Introduction

The retinoblastoma tumor suppressor protein (RB1) regulates G1/S-phase cell cycle progression and is a critical mediator of antiproliferative signaling. Although phosphorylation plays an important role in the functional regulation of RB1, the transcriptional regulation of RB1 is much less known [1]. RB1-inducible coiled-coil 1 (RB1CC1: the symbol used here, which is approved by the Human Genome Organization [HUGO] Gene Nomenclature Committee; it is also known as FIP200, focal adhesion kinase family-interacting protein of 200 kDa) was identified as an RB1 pathway regulator that in particular enhances *RB1* transcription [2],[3]. Recently, we have demonstrated that nuclear RB1CC1 binds to a GC-rich region 201bp upstream (from the initiation ATG) of the RB1 promoter and activates the expression of RB1 [4]. A genetic rearrangement of *RB1CC1* has also been suggested to be involved in the tumorigenesis of breast cancer [3], [5]. Interestingly, it has been reported that RB1CC1 binds and stabilizes p53 [6], suggesting that RB1CC1 might be an important mediator connecting the RB1 and p53 pathways. Our present study investigates the mechanism of RB1CC1 enhancement of the RB1 pathway. RB1CC1 forms a complex with p53 and/or hSNF5, a chromatin-remodeling factor, in cell nuclei, and activates the transcription of *RB1*, *p16* and *p21*. In addition, nuclear RB1CC1 significantly correlates RB1 and p16 expression in breast cancer tissue *in vivo*.

## Results

### RB1CC1 forms a complex with hSNF5 and/or p53

**Figure 1:**
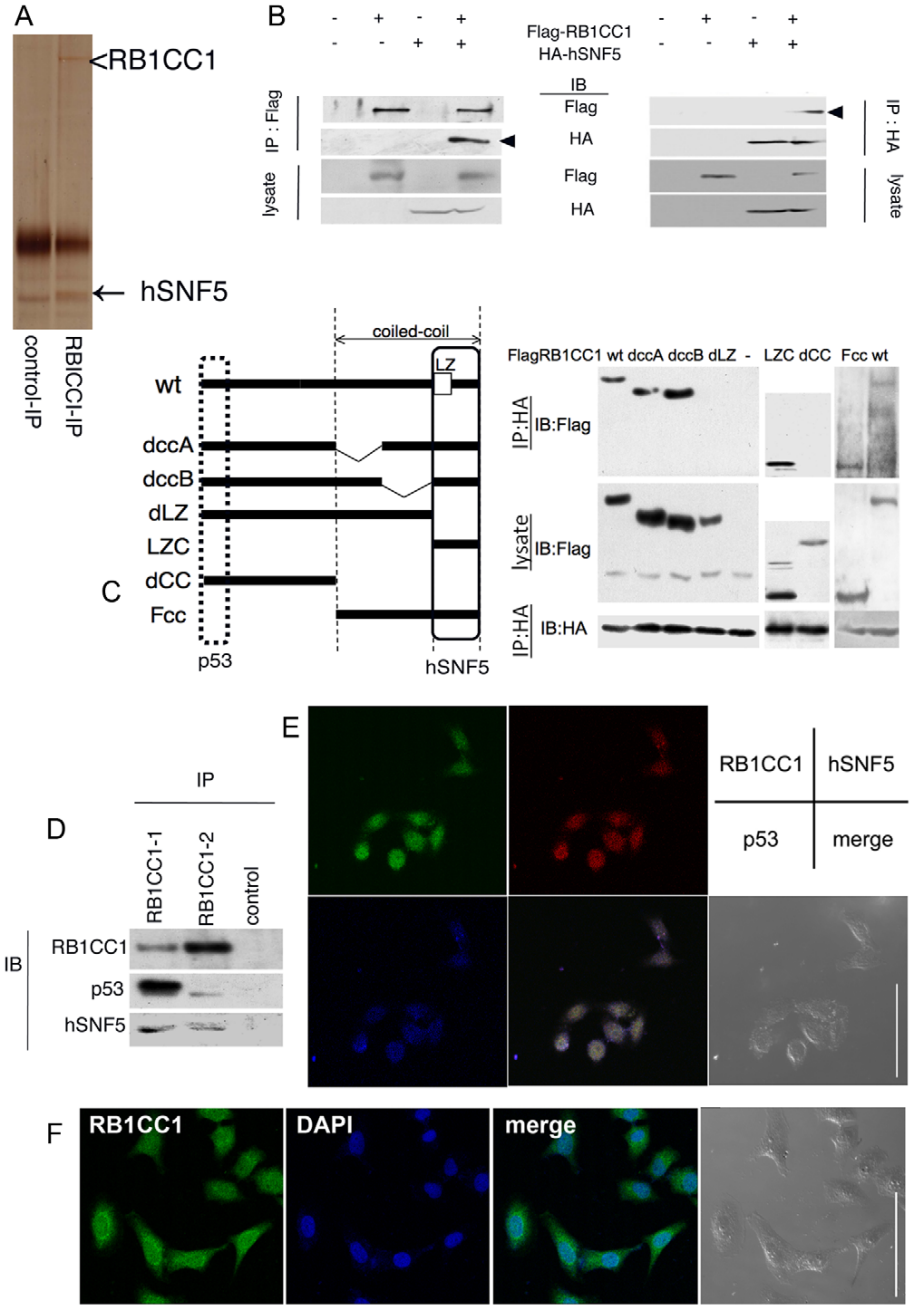
RB1CC1 forms a complex with hSNF5 and/or p53. (**A**) MCF-7 cell lysates were immunoprecipitated (IP) with anti-RB1CC1 antibody and analyzed by SDS-PAGE followed by silver staining. The molecule that co-immunoprecipitated with RB1CC1 was identified as hSNF5 by liquid chromatography-tandem mass spectrometry (LC-MS/MS). (**B**) Flag-RB1CC1 and HA-hSNF5 were co-transfected into HEK293 cells. Lysates were immunoprecipitated with anti-Flag or anti-HA antibody, and analyzed by immunoblots as indicated. Arrowheads indicate the co-immunoprecipitated signals. (**C**) HEK293 cells were transfected with both HA-hSNF5 wildtype (full) and Flag-RB1CC1 variants; wildtype (wt), dccA, dccB, dLZ, LZC, dCC and Fcc. Schematic diagrams of RB1CC1wt and mutants are also indicated on the left side. The rectangle shows the binding site for hSNF5, and the broken rectangle indicates that for p53. Cell lysates were immunoprecipitated with anti-HA antibody and immunoblotted as indicated. (**D**) MCF-7 lysates were immunoprecipitated with two kinds of anti-RB1CC1 antibody and analyzed by immunoblots. (**E**) RB1CC1 (green), hSNF5 (red), p53 (blue) and the merged image in HeLa cells. Each protein is immunofluorescently labeled by Alexa 488, 594 or 350 respectively. Scale bar, 50 µm. (**F**) RB1CC1 (green), DAPI and the merged image in HeLa cells. RB1CC1 is immuno-labeled by Alexa 488, and DAPI is displayed with blue fluorescence in cell nuclei. Scale bar, 50 µm.

We initially attempted to identify RB1CC1-binding proteins in MCF-7 breast cancer cells by an immunoprecipitation assay followed by liquid chromatography-tandem mass spectrometry (LC-MS/MS). A chromatin remodeling factor, hSNF5 (also known as BAF47 or INI1) was co-immunoprecipitated with RB1CC1 (Fig. 1A). The pull-down assay using RB1CC1 deletion-mutants revealed that the C-terminal region of RB1CC1 (aa 1364–1594) was required for the interaction with hSNF5 (Fig. 1B and C). The N-terminus of RB1CC1 also interacts with p53 [6], so we considered that RB1CC1 might form a complex with hSNF5 and/or p53 to inhibit tumor growth. The complex formation between RB1CC1, hSNF5 and p53 was evaluated by immunoprecipitation and immunofluorescence assays. RB1CC1, hSNF5 and p53 were co-immunoprecipitated with two kinds of antibodies recognizing different sites (aa. 25–271 and 549–817) of RB1CC1; the composition of each of the precipitates is different, probably due to the preferable fractions to each antibody (Fig. 1D). RB1CC1-1 and -2 antibodies have tended to bind more preferably to the nuclear and cytoplasmic fractions, respectively. hSNF5 and abundantly nuclear p53 (that might be more phosphorylated and slowly mobilized in PAGE) might be preferably co-precipitated with the complex between nuclear RB1CC1 and RB1CC1-1 antibody. Reciprocally, RB1CC1-2 antibody might dominantly bind to abundant cytoplasmic RB1CC1 as well as hSNF5 and a few of p53 in cytoplasm. RB1CC1, hSNF5 and p53 were co-localized in cell nuclei with the exception of nucleoli (Fig. 1E and F; Fig. S1). The data indicated that RB1CC1 forms a complex with hSNF5 and/or p53 mainly in cell nuclei.

### RB1CC1 binds to and activates the promoters of RB1, p16 and p21, cooperating with p53 and/or hSNF5

**Figure 2:**
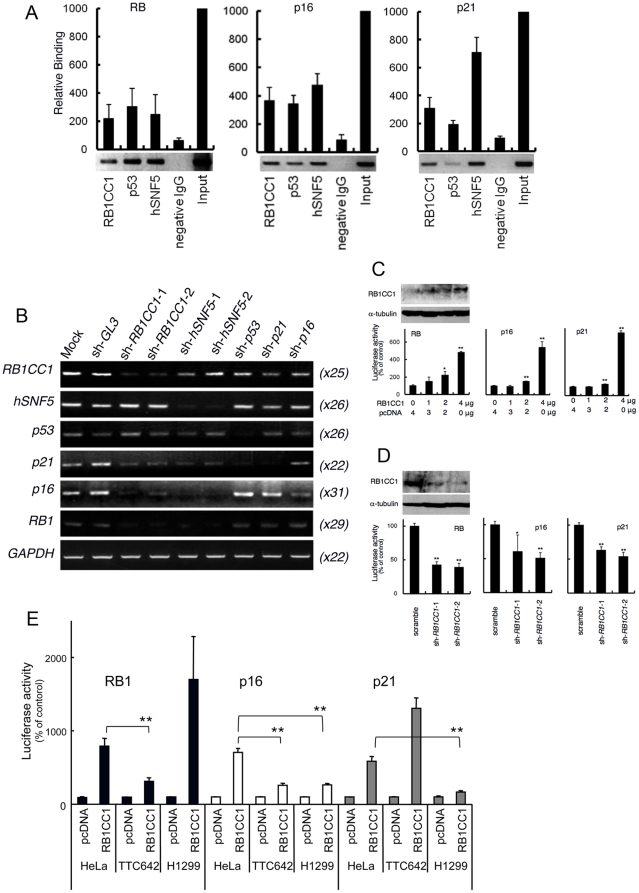
RB1CC1 binds to and activates the promoters of *RB1*, *p16* and *p21*, cooperating with p53 and/or hSNF5. (**A**) Chromatin immunoprecipitation (ChIP) assay was performed in HeLa cells. The chromatin was immunoprecipitated by anti-RB1CC1, anti-p53, or anti-hSNF5 antibody. Each precipitated DNA was analyzed by quantitative and semi-quantitative PCR using the promoter-specific primers for *RB1*, *p16* and *p21*. The signal intensities were defined based on those originating from input DNA (2ng/µl) of HeLa cells, which were set as 1,000. (**B**) Semi-quantitative RT-PCR was performed at the individual cycle number for each transcript in HeLa cells treated with shRNA for *RB1CC1*, *hSNF5* or *p53*. Sh-*GL3*, sh-*p21* and sh-*p16* were used as controls. Parentheses indicate the PCR cycle numbers. (**C**–**D**) In HeLa cells, luciferase activity of the promoter for *RB1*, *p16* and *p21* increased significantly upon transfection of plasmid expressing RB1CC1 (RB1CC1wt) (*C*) and decreased markedly upon knockdown of RB1CC1 using sh-*RB1CC1*-1 and -2 (*D*), compared with cells transfected with pcDNA or sh-RNA scramble, respectively, as controls. The values indicate the means ± standard errors from quadruplicate experiments. (Student's t-test; asterisks (*) indicate statistically significant differences between pcDNA and RB1CC1wt or between scramble and sh-*RB1CC1*. *, p<0.05; and **, p<0.01). The efficiency of over-expression or knockdown of RB1CC1 was validated by Western blots as indicated in the upper panels. (**E**) After the introduction of RB1CC1wt, luciferase activities of the promoters for *RB1*, *p16* and *p21* were compared among HeLa, TTC642 (hSNF5-null) and H1299 (p53-null) cells. Asterisks (**) indicate statistically significant differences (Student's t-test; p<0.01) between HeLa and TTC642 or between HeLa and H1299.

RB1CC1 [2], [4], [6] and hSNF5 [7], [8], [9], [10] enhance transcription of the molecules involved in the RB1 pathway, so we examined whether RB1CC1, hSNF5 and p53 bind to the promoter regions of *RB1*, *p16* and *p21*. Chromatin immunoprecipitation (ChIP) assays using antibodies against RB1CC1, hSNF5 and p53 indicated that promoter fragments of *RB1*, *p16* and *p21*, which were amplified from HeLa cell chromatin, were precipitated by each antibody (Fig. 2A), suggesting that the molecules are recruited to the promoter regions of the RB1 pathway. The involvement of these three molecules in the expression of *RB1*, *p16* and *p21* were validated by introducing sh-RNA for *RB1CC1* (sh-*RB1CC1*), *hSNF5* (sh-*hSNF5*) and *p53* (sh-*p53*) into HeLa cells. The knockdown effects on *RB1CC1*, *hSNF5*, *p53*, *p21*, *p16* and *RB1* mRNA expression were analyzed by semi-quantitative RT-PCR. sh-*RB1CC1* and sh-*hSNF5* decreased mRNA levels of *RB1*, *p16* and *p21*, whereas sh-*p53* decreased only *p21* mRNA (Fig. 2B). In other words, RB1CC1 and hSNF5 are required to maintain transcriptions of *RB1*, *p16* and *p21*, whereas p53 is specifically required for transcription of *p21*. RB1CC1 significantly activated the promoters of *RB1*, *p16* and *p21* (Fig. 2C), and the knockdown of RB1CC1 suppressed them (Fig. 2D). These promoter enhancements by RB1CC1 were compared among HeLa, TTC642 (hSNF5-null) and H1299 (p53-null) cells, in order to validate the requirement for hSNF5 or p53 when RB1CC1 activates *RB1*, *p16* and *p21*. RB1CC1 was unable to provide significant enhancement of *RB1* and *p16* promoters in TTC642 cells, and played only a small role in *p16* and *p21* transcription in H1299 cells (Fig. 2E). Considering the RB1 pathway initiated by RB1CC1, RB1CC1 requires hSNF5 for enhancement of *RB1* and p53 for enhancement of *p21* transcriptions, but both hSNF5 and p53 are needed for RB1CC1 enhancement of *p16* transcription. In summary, RB1CC1 requires interaction with both hSNF5 and p53 to provide a strong activation of *RB1*, *p16* and *p21* promoters.

### RB1CC1 activates the RB1 pathway and suppresses tumor growth

**Figure 3:**
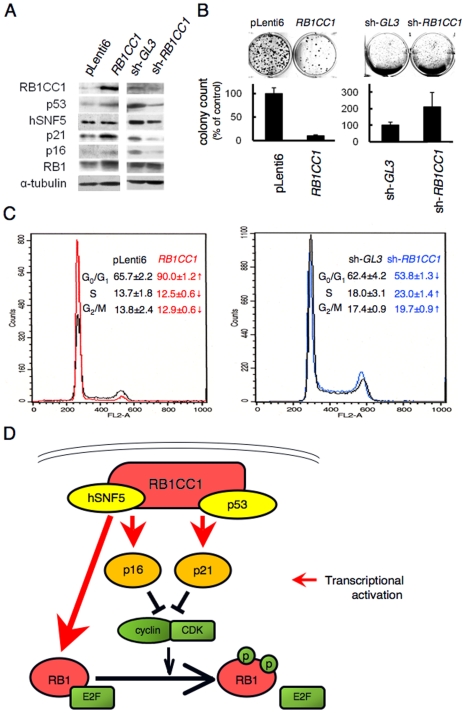
RB1CC1 activates the RB1 pathway and suppresses tumor growth. (**A**) HeLa cells were lentivirally transduced with *RB1CC1* or sh-*RB1CC1*. Lysates were immunoblotted as indicated. (**B**) Growth assays in HeLa cells transduced as in *a*. The data were quantified from triplicate experiments (lower column). Representative immunoblots, plates and mean colony numbers ± standard errors are shown. (**C**) After the lentiviral modification of RB1CC1 expression, NIH3T3 cells were analyzed by flow-cytometry. *RB1CC1* and sh-*RB1CC1* are indicated by red (left) and blue (right), respectively. The data (mean percentages ± standard deviations) were confirmed in triplicate experiments, and a representative flow cytometric graph is demonstrated. pLenti6 and sh-GL3 were used as controls. Arrows indicate that increases (up-arrow) and decreases (down-arrow) were statistically significant according to the Student's t-test; p<0.05 (**D**) RB1CC1, hSNF5 and p53 form the transcriptional complex in cell nuclei, and activate *RB1*, *p16* and *p21*.

To evaluate whether RB1CC1 enhances RB1 pathways to suppress cancer cell growth, HeLa cells were lentivirally transduced with *RB1CC1* cDNA or shRNA. Ectopic expression of RB1CC1 increased the protein levels of p53, p21, p16 and RB1, and inhibited the cell growth (Fig. 3A and B, left panel). In contrast, the knockdown of endogenous RB1CC1 by sh-*RB1CC1* decreased the levels of p53, hSNF5, p21 and p16, and enhanced the cell growth (Fig. 3A and B, right panel). The same experiments in two breast cancer cell lines (MCF-7 and SK-BR3) yielded similar results (Fig. S2A-D). In *RB1CC1*-transduced cells, the number of G0/G1-phase-cells was increased concomitantly with the decrease of the numbers of cells in S and G2/M phases. Introduction of sh-*RB1CC1* decreased the G0/G1 population and increased the populations of cells in S and G2/M (Fig. 3C; Fig. S2E).

Taken together, the above-mentioned data indicated that RB1CC1 formed a complex with hSNF5 and/or p53, and globally activated the transcription of *RB1*, *p16* and *p21* (Fig. 3D).

### Expressional analysis of the molecules involved in the RB1CC1-RB1 pathway in breast cancers *in vivo*

**Figure 4:**
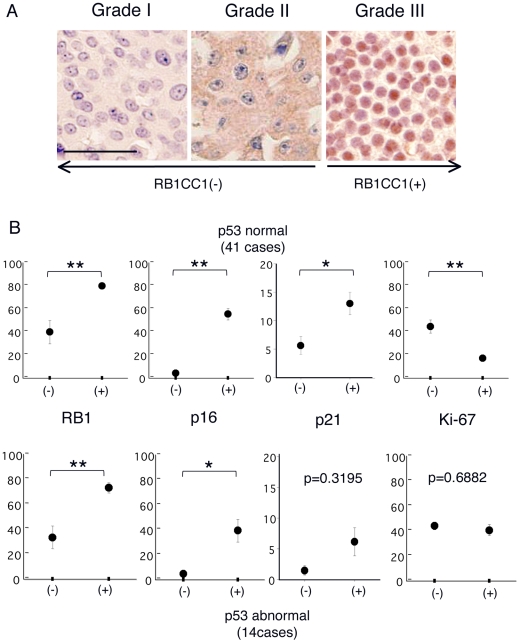
*In vivo* significance of RB1CC1. (**A**) The immunohistochemical grades of RB1CC1 were classified as I–III: I, negative stain in both cytoplasm and nuclei; II, positive stain only in cytoplasm and negative in nuclei; III, positive stain in nuclei and/or cytoplasm. Grades III, expressing RB1CC1 abundantly in cell nuclei, were recorded as RB1CC1 (+), whereas grades I–II without nuclear RB1CC1 were recorded as RB1CC1 (−). Scale bar shows 50µm. (**B**) Correlation between RB1CC1 and RB1, p16, p21 or Ki-67 in p53normal (upper graphs) and abnormal (lower graphs) cases. RB1CC1 (−) or (+) status is indicated on the horizontal line, and each labeling index is indicated on a vertical line in the graphs. An abnormal p53 status was defined as a case of breast cancer when more than 50% of tumor cells were strongly positive for p53, while less than 10% of the cells were positive for p21. Such case highly indicated that mutant p53 protein was accumulated, and that the intact functions of p53 (like transcriptional activation for *p21*) were loss. Student's *t*-test was used to evaluate the correlations (*, p<0.05; and **, p<0.01).

To evaluate the correlation between the proliferative activity and the above-mentioned RB1CC1 pathway of RB1 activation involving p53 and hSNF5 in tumor tissue *in vivo*, we evaluated the expressional status of RB1CC1, RB1, p16, p21, Ki-67, p53 and hSNF5 in 59 cases of breast cancer. Four cases of RB1 nullizygotes indicated a high Ki-67 proliferation index (means ± standard errors = 69.7±6.6%). The immunoreactivity of p53—referring to p21 expression—was divided into two categories, “normal” (p53nor) for weak staining and “abnormal” (p53ab) for strong staining, which highly indicated that mutant p53 protein was accumulated. There were 41 and 14 cases of p53nor and p53ab, respectively, in this series. hSNF5 was highly maintained in breast cancers, irrespective of RB1CC1 or p53 status (data not shown). The immunohistochemical results for RB1CC1 were quantitatively graded as I-III. Grade III, expressing RB1CC1 abundantly in cell nuclei, was recorded as RB1CC1 (+), whereas grades I–II without nuclear RB1CC1 were recorded as (−) (Fig. 4A). As reported previously [4], the expression of RB1 was quite well correlated with RB1CC1 expression in the cases recorded as RB1CC1 (+) and was higher than in the RB1CC1 (−) cases (Fig. 4B, left). p16 expression was also correlated positively with nuclear RB1CC1 expression (Fig. 4B, 2^nd^ left). Labeling indices of p21 and Ki-67 in p53nor cases - but not in the p53ab cases - were well correlated with the expression status of RB1CC1 (Fig. 4B, 3^rd^ left and right). Together, the expression status of RB1CC1 and p53 were associated with increased expressions of RB1, p16 and p21, which in turn influenced the Ki-67 index of proliferation activity in clinical breast cancers. Therefore, the immunohistochemical status of RB1 and p53 as well as that of RB1CC1 should be good predictors of the proliferative activity; thus, the RB1CC1-RB1 pathway demonstrated in our experiments could play a significant role in the progression of clinical breast cancer.

## Discussion

RB1CC1 is a novel regulator of RB1 that dephosphorylates RB1 [2], [6] and increases its expression [2], [4]; in addition, a genetic rearrangement of *RB1CC1* might be involved in breast cancer tumorigenesis [3], [5]. More recently, we reported that nuclear RB1CC1 directly activates the *RB1* promoter through a 201bp upstream GC-rich region (−201/U-GCbox) [4]. To clarify more precisely the molecular mechanism of the RB1CC1 action, an immunoprecipitation assay followed by LC-MS/MS was attempted, and a chromatin remodeling factor, hSNF5 was identified. RB1CC1 also interacts with p53, so RB1CC1 is thought to form a complex with hSNF5 and/or p53. It is thought that RB1CC1 induces *RB1* [2], [4], and that hSNF5 enhances *p16* (also known as INK4a or CDKN2A) and/or *p21* (also known as CIP1, WAF1 or CDKN1A) [7], [8], [9], [10]. In fact, immunoprecipitation and immunofluorescence assays have indicated that a complex between RB1CC1, hSNF5 and p53 forms in cell nuclei, and ChIP, quantitative RT-PCR and the luciferase assays for *RB1*, *p16* and *p21* have suggested that the coordinated transcriptional enhancements of these molecules are affected by the complex including RB1CC1, hSNF5 and p53. However, RB1CC1, hSNF5 and p53 are not equally important to the stimulation of each of the promoters of *RB1*, *p16* or *p21*, while three molecules are recruited to each promoter. For example, silencing *p53* in HeLa cells had no effect on the transcription of *RB1*, and RB1CC1 over-expression can activate the *RB1* promoter in H1299 (p53-null) cells. These mean that p53 isn't essentially required to *RB1* transcription, and that RB1CC1 and hSNF5 can co-operate to activate *RB1* transcription. Thus, all three molecules are not always required for activation of each of the promoters, while all three are needed for the coordinated transcriptions of *RB1*, *p16* and *p21*. We have also established earlier that nuclear RB1CC1 binds to and activates the *RB1* promoter [4]. Interestingly, a point-mutation in the −201/U-GCbox of the *RB1* promoter was identified in a hereditary retinoblastoma family [11]. It should be pointed out that the −201/U-GCbox of *RB1* and the binding transcriptional complex that includes RB1CC1, hSNF5 and p53 play important roles in human carcinogenesis.

In the present study, RB1CC1, in collaboration with hSNF5 and p53, activates the RB1 pathway and suppresses tumor cell growth. In order to activate the RB1 pathway effectively, RB1CC1 must be expressed in cell nuclei and should form a complex with hSNF5 and/or p53, as suggested in the immunocytochemical and immunohistochemical data of this study. PIASy (a protein-inhibitor of activated STAT protein y) interacts with the C-terminus (aa 1350–1594) of RB1CC1 and recruits an interacting complex between PIASy and RB1CC1 from cytoplasm into nuclei [12]. In addition, PIASy negatively regulates mammalian target of rapamycin (mTOR) activity stimulated by cytoplasmic RB1CC1, and positively activates the p53-p21 signaling pathway together with nuclear RB1CC1 [12]. These data seem to be compatible with our presented data on the sublocalization of the complex composed of RB1CC1, hSNF5 and p53. The nuclear molecular complex, including RB1CC1, hSNF5, p53 and additional PIASy, can form a large transcriptional component to activate the RB1 pathway coordinately and repress the tumorigenesis in human breast tissue.

Nuclear expression of RB1CC1 could be important for tumor suppression. As reported previously, RB1CC1 is located not only in the nucleus but also in the cytoplasm [4], [6], [13], [14]. Cytoplasmic RB1CC1 has been suggested to be the equivalent of yeast Atg17, and several studies have indicated that RB1CC1 functions as an essential molecule in autophagy regulation [13], [14], [15]. Autophagy has been implicated in tumorigenesis, but its precise role is ambiguous. It is conceivable that autophagy has different roles in the different stages, or contexts, of tumorigenesis. Young, et al. [16] have reported that autophagy mediates the mitotic senescence, a window into early tumor development. We suggest that cytoplasmic-nuclear transition of RB1CC1 plays a key role in autophagy-senescence association. Our data *in vitro* and *in vivo* suggest that RB1CC1 plays different roles according to its subcellular location. Nuclear RB1CC1 forms a complex with hSNF5, p53 and any other component that contributes to the transcription of the RB1 pathway, indicating a possible linkage to mitotic senescence. However, cytoplasmic RB1CC1 seems to play no role as a tumor suppressor activating the RB1 pathway.

Nuclear RB1CC1 expression significantly correlated with those of RB1 and p16 in breast cancer tissue *in vivo*, irrespective of p53 status. Ki-67 proliferation index and p21 expression were affected by both RB1CC1 and p53, and the Ki-67 index also depended on RB1 status. The expressions of RB1, p16 and p21 affect the Ki-67 index of proliferative activity, so the immunohistochemical status of RB1 and p53 as well as that of RB1CC1 can predict the proliferative activity of the tumor. Thus, the present data suggested that progression of the breast cancers was under the strong influence of RB1CC1, as well as RB1 and p53 status. We expect that the combined immunohistochemical evaluation of RB1, RB1CC1 and p53 will provide insight into their clinical applications, such as possible use as prognostic biomarkers.

In summary, we have reported here that RB1CC1, connecting the RB1 and p53 pathways as a possible mediator, forms a complex with hSNF5 and/or p53 that enhances the RB1 pathway globally. Immunohistochemical evaluation of RB1 and p53 in combination with RB1CC1 may be a useful biomarker in convenient, routine clinical assays and provide a prediction of the biological behavior of breast neoplasms and/or the tumors of other organs.

## Materials and Methods

### Cell culture

TTC642 was kindly provided by Drs. Shigeru Ohta, Hiroyuki Shimada and Timothy J. Triche (Childrens Hospital Los Angeles, Los Angeles, CA). HeLa, HEK293, MCF-7, SK-BR3 and H1299 cells were purchased from American Type Culture Collection (MD), and cultured in Dulbecco's modified Eagle's medium (DMEM) or RPMI 1640 containing 10% fetal bovine serum. All cell culture media were supplemented with penicillin (50 units/ml) and streptomycin (50 mg/ml).

### Antibodies and reagents

Rabbit antisera against RB1CC1 (aa. 25–271, 549–817 as each epitope) were generated as previously reported [4], [17], and the purified IgG were used in the experiments. Anti-p53 (FL-393) and anti-p21 (F-5) were obtained from Santa Cruz Biotechnology (CA). Anti-HA (3F10) was from Roche (Germany). Anti-Flag (M2) and anti-tubulin α (DM 1A) were purchased from Sigma (MO). The other antibodies were from BD Biosciences (CA).

### Immunoprecipitation of protein complex followed by liquid chromatography- tandem mass spectrometry (LC-MS/MS)

The MCF-7 breast cancer cells were lysed in EBC lysis buffer containing 50 mM Tris-HCl (pH 8.0), 120 mM NaCl, 1 mM EDTA, 0.5% Nonidet P-40 (NP-40), 0.25% sodium deoxycholate, 0.2 mM Na_3_VO_4_, 100mM NaF and 1% protease inhibitor cocktail (Wako Chemicals, Japan), and then centrifuged for 20 min at 20,000×g at 4°C. The 5 ml supernatant (∼10mg/ml) was incubated with 100 µg of anti-RB1CC1 purified IgG primary antibody at 4°C overnight. The immunocomplexes were bound to protein G-agarose (Pierce, IL) for additional 2 h at 4°C and washed five times with NETN (20mM Tris-HCl (pH 8.0), 100 mM NaCl, 1 mM EDTA, 0.5% NP-40). The proteins bound to the protein G-Sepharose were eluted by adding Laemmli SDS sample buffer containing 62.5 mM Tris-HCl (pH 6.8), 10% glycerol, 5% 2-mercaptoethanol, 2% SDS, and 0.01% bromophenol blue to the gel and boiling it for 3 min. After centrifugation at 10,000×g for 2 min, the supernatant was subjected to SDS-polyacrylamide gel electrophoresis (SDS-PAGE), and the separated proteins were visualized by silver staining. The strongly stained specific band was in-gel digested with trypsin, and the recovered peptides were analyzed using an electrospray ion-trap mass spectrometer (LCQ, Finnigan MAT, CA) coupled online with capillary HPLC (Magic 2002, Michrom BioResorces, CA) to acquire mass spectrometry/mass spectrometry (Ms/Ms) spectra. Data derived from the Ms/Ms spectra were used to search a compiled protein database, which was composed of database NR and a six-reading-frame translated EST database, to identify the protein by using the publicly available program PROWL (http://prowl.rockefeller.edu).

### Plasmid DNA and gene transfer

External and internal deletion mutants for *RB1CC1* (GenBank no. NM_014781) were generated in the pcDNA3.1 plasmid vector (Invitrogen), by a combination of PCR-based manipulations with appropriate external primers at the positions described below and restriction enzyme digestion. The nucleotides of all constructs were confirmed by DNA sequencing. The *RB1CC1* mutants dccA, dccB, dLZ, LZC, dCC and Fcc deleted the amino acids 863–1083, 1078–1363, 1364–1594, 1–1356, 824–1594 and 1–863, respectively (Fig. 1C, left panel). Transfection was performed using FuGENE HD (Roche) according to the supplier's recommendations. Plasmid vectors of RNAi for *RB1CC1* were prepared as described previously [17]. Briefly, two kinds of lentiviral sh-RNA vectors (sh-*RB1CC1*-1 and -2) corresponding to different target sites (nt. 2070–2090 and nt. 4611–4631, respectively) on *RB1CC1* mRNA (GenBank no. NM_014781) were synthesized in vitro by inserting artificial oligonucleotides into the pSIH1-H1-Puro sh-RNA vector (System Biosciences, CA). Vectors for sh-RNA of *hSNF5* and *p53* were prepared by OpenBiosystems (AL). For non-silencing controls, sh-*GL3* and scramble vectors were purchased from System Biosciences and OpenBiosystems, respectively. Additionally, human *RB1CC1* cDNA was subcloned into a pLenti6 vector (Invitrogen). Lenti-virus transferring sh-RNA or cDNA was prepared with each packaging mix, according to each manufacturer's instructions. Cloned and expanded cells were selected in the presence of puromycin or blasticidin, respectively, and used in the experiments.

### Pull-down assay

Flag-tagged-wildtype (wt) *RB1CC1* or its mutants (dccA, dccB, dLZ, LZC, dCC and Fcc) were transfected into HEK293 cells combined with HA-tagged hSNF5 (full). The cells were lysed in TNE buffer (20 mM Tris-HCl, 150 mM NaCl, 5 mM EDTA, 1% NP-40 and 1 mM Na_3_VO_4_ containing a mixture of protease inhibitors). The lysates were centrifuged, and the supernatants were incubated with anti-Flag or anti-HA immobilized beads for 2 h at 4°C. The beads were washed five times with TNE buffer and boiled in the presence of Laemmli SDS sample buffer. The protein complexes were resolved by SDS-PAGE, transferred to PVDF membrane filters and subjected to immunoblot analysis with the indicated antibodies.

### Immunoprecipitation to detect the protein complex

MCF-7 monolayer cells were rinsed with ice-cold phosphate-buffered saline (PBS) and scraped into ice-cold NP-40 lysis buffer (20 mmol/L Tris (pH 8.0), 137 mmol/L NaCl, 1% NP-40, 10% glycerol) supplemented with 1% protease inhibitor cocktail (Wako Chemicals) and 3 mmol/L Na_3_VO_4_. Lysates were incubated on ice for 20 minutes, cleared by centrifugation, and protein concentration was determined by the bicinchoninic acid assay (Pierce). Solutions containing 400 to 1,000 µg of protein were briefly precleared with protein-G-agarose beads (Pierce) and then used for the immunoprecipitation reaction with 2 kinds of anti-RB1CC1 rabbit polyclonal IgGs or normal control rabbit IgGs at 4°C overnight, followed by incubation with protein-G beads for 2 hours to collect immune complexes. Finally, the beads were washed with NP-40 buffer five times, resuspended in SDS sample buffer, boiled, resolved on SDS-PAGE, and analyzed by immunoblots as described below.

### Immunoblot analysis

Cells were generally lysed for Western blotting as described by Sarbassov, et al [18]. After clearing lysed materials by centrifugation at 15,000×g for 1 min, the supernatants were boiled in SDS sample buffer. Proteins resolved by SDS-PAGE were transferred to polyvinylidene difluoride (PVDF) filters. After blocking of the filters with TBS-T (10 mM Tris-HCl (pH 7.6), 150 mM sodium chloride, 0.1% Tween 20) containing 5% bovine serum albumin (BSA), the filters were incubated overnight with the indicated primary antibodies in TBS-T containing 2% BSA at 4°C. The filters were then washed in TBS-T and incubated for 1 h in horseradish peroxidase-conjugated anti-mouse, anti-rabbit, or anti-rat IgG (GE Healthcare, UK) diluted 1∶20,000 in TBS-T containing 2% BSA. After several washes with TBS-T, the immunoreactivity was detected using the ECL system (GE Healthcare) according to the procedures recommended by the manufacturer.

### Immunofluorescence assay

MCF-7 and HeLa cells were cultured on LabTek™ chamber slides (BD Biosciences) to 70% confluence, fixed with 1% buffered formaldehyde and 70% ethanol, and then permeabilized with 0.1% Triton X-100. The primary rabbit anti-RB1CC1 IgG, mouse anti-hSNF5 IgG2a (BAF48), and rabbit anti-p53 IgG (FL393) were conjugated with Alexa 488, 594 and 350 by the ZenonTM labeling kit (Molecular Probes). To evaluate the subcellular location, 4′,6-diamidino-2-phenylindole (DAPI) was also applied in HeLa and MCF-7 cells. The labeled primary antibodies were incubated overnight at 4°C. The slides were then washed with PBS to remove excess fluorescent dye and mounted with glycerol. The specimens were observed and photographed under identical conditions using a confocal laser-scanning microscope (C1si, Nikon Co.).

### Chromatin immunoprecipitation (ChIP) assay

For ChIP of the endogenous *RB1*, *p21* and *p16* promoter regions in HeLa cells, the ChIP-IT™ control kit and ChIP-IT™ Express (Active Motif, CA) were used, and the assay performed essentially as described in the Active Motif protocol. The used antibodies were anti-RB1CC1, anti-hSNF5 (BAF48), anti-p53 (FL-393) and control rabbit IgG (Active Motif). The immunoprecipitated DNA was quantified by the quantitative (Real-time) and semi-quantitative polymerase chain reaction. The amplified promoter regions of *RB1* and *p16* were from −385 to −110, and from −712 to −529, respectively (the first ATG was defined as +1). For the *p21* promoter, the region from −2432 to −2175 (the transcriptional start site was defined as +1) was amplified. The sequences of the PCR primers and suitable PCR conditions are available upon request.

### Reverse transcription-polymerase chain reaction (RT-PCR) in the specifically knocked-down cells for RB1CC1, hSNF5 or p53

Transcriptional expressions of *RB1*, *p21* and *p16* were evaluated by the semi-quantitative reverse transcription-polymerase chain reaction (RT-PCR) method between sh-*GL3* control and the gene-specific knocked-down HeLa cells, transfected lentivirally with shRNA for *RB1CC1* (sh-*RB1CC1*), *hSNF5* (sh-*hSNF5*) and *p53* (sh-*p53*). Transcribed mRNA in each knocked-down cell was adequately quantified by semi-quantitative RT-PCR at the individual cycle number for each transcript. The sequences of the PCR primers and suitable RT-PCR conditions are available upon request.

### Luciferase-reporter assay

HeLa, TTC642 (hSNF5-null) and H1299 (p53-null) cells were seeded at 0.7×10^5^ cells/well in 6-well plates. On the next day, *RB1*-, *p21*-, or *p16*-luciferase reporter plasmid was cotransfected by FuGENE HD (Roche) with plasmids empty vector and/or *RB1CC1*-cDNA, -shRNA vector. Twenty-four hours later, cells were lysed, and luciferase activity was analyzed using a Luciferase assay kit (Toyo Ink, Japan) and luminometer (EG&G Berthold Lumat LB 9507). The *p16* promoter region from −900 to −22 (the first ATG was defined as +1) was contained in the luciferase reporter plasmids (pGL3, Promega, WI). *RB1*- and *p21*-luciferase plasmids were constructed as reported previously [2], [19].

### Cell cycle analysis

To analyze the cell cycle between pLenti6 control and RB1CC1-expression; sh-*GL3* control and RB1CC1-knockdown cells, the distribution of DNA content was measured using the CycleTEST™ PLUS kit (Becton Dickinson). Flow cytometric data were collected on a FACSCaliber, and analyzed with CellQuest software.

### Cell growth assay

The cells were prepared at 1×10^5^ cells/well in 6-well plates, lentivirally transduced with cDNA or shRNA for *RB1CC1*, and incubated in the presence of blasticidin or puromycin, respectively. After 10–20 days of transduction, the cells were fixed with 10% acetate/10% methanol for 20 minutes, and stained with 0.4% crystal violet in 20% ethanol for 15 minutes to visualize the colonies.

### Patients and histology

A total of 72 consecutive patients with operable primary breast cancers, treated in Shiga University of Medical Science or in Osaka Medical Center for Cancer and Cardiovascular Diseases around 1999, were studied. Among these, we failed to assess the immunohistochemical evaluations in 13 tumor specimens as a result of tissue loss during slide preparation. Therefore, specimens from 59 patients with primary breast cancer were analyzed in this study. They were collected with the written informed consent of patients, and after approval by the Ethics Committee of Shiga University of Medical Science. The pathological diagnoses of all the specimens were confirmed by at least two surgical pathologists, and classified according to the World Health Organization (WHO) guidelines.

### Immunohistochemistry

To evaluate RB1CC1, RB1, p16, p21, Ki-67, p53 and hSNF5 in human breast cancer tissues, surgical specimens from 59 cases were transferred to 10% buffered formalin and fixed overnight. The fixed samples were embedded in paraffin and serially sliced into 5-µm sections. Deparaffinized sections were autoclaved at 120°C for 1 min, immersed in 0.3% H_2_0_2_ and rinsed with 1xPBS before incubation overnight at 4°C with each of the primary antibodies. The sections were rinsed with 1xPBS and incubated with the secondary antibody (Simple Stain MAX-PO; Nichirei, Japan) at room temperature for 1 hour. The sections were then stained with 3,3′-diaminobenzidine tetrahydrochloride (DAB), and counter-stained with hematoxylin.

### Microscopic evaluation

At first, we had classified into 3 categories: I, negative stain in both cytoplasm and nuclei; II, positive stain only in cytoplasm and negative in nuclei; III, positive stain in nuclei and/or cytoplasm. However, our recent study proved that RB1 expression was significantly higher in the cases with nuclear RB1CC1 expression (grade III) than in the cases without nuclear RB1CC1 (grade I and II) [4]. Therefore, RB1CC1 staining grades I-II and III were defined as -negative (−) and -positive (+), respectively. A dysfunctional status of p53 was assessed immunohistochemically by the percentage of cells that were positive for p53 and p21. An abnormal p53 status (p53ab) was defined as a case of breast cancer when more than 50% of tumor cells were strongly positive for p53, while less than 10% of the cells were positive for p21. Nuclear expressions of p16 and p21 in tumor cells were recognized as positive labels.

### Statistical analysis of difference between RB1CC1 and the other indicators

Student's *t*-test was used to evaluate the relationships between RB1CC1 staining and the labeling indices of RB1, p16, p21 and Ki-67. A p-value of <0.05 was considered statistically significant.

## Supporting Information

Figure S1 RB1CC1 interacts with hSNF5 and p53 mainly in cell nuclei. (A) RB1CC1 (green), hSNF5 (red), p53 (blue) and the merged image in MCF-7 cells. Each protein is immunofluorescently labeled by Alexa 488, 594 or 350 respectively. Scale bar, 50µm. (B) RB1CC1 (green), DAPI and the merged image are evaluated in MCF-7 cells. RB1CC1 is immuno-labeled by Alexa 488, and DAPI is displayed with blue fluorescence in cell nuclei. Scale bar, 50µm.
(0.90 MB TIF)

Figure S2 Modulation of RB1CC1 affects the growth of breast cancer cells through RB1, p21 and/or p16 expression. (A) In MCF-7 breast cancer cells, RB1CC1 induced RB1, p53 and p21 expression, and, conversely, the knockdown (sh-RB1CC1) reduced their expression. (B) RB1CC1 suppressed and sh-RB1CC1 enhanced the cell growth of MCF-7. (C) In SK-BR3 breast cancer cells, RB1CC1 induced RB1, hSNF5, p16 and p21 expression. sh-RB1CC1 decreased the p16 and p21 contents. (D) RB1CC1 suppressed and sh-RB1CC1 enhanced the cell growth of SK-BR3. A and C indicate the Western blots data. B and D show the results of cell growth assays. Modulation was performed by lentiviral transduction of cDNA or sh-RNA of RB1CC1 into the cells. pLenti6 and sh-GL3 were used as controls. The experiments were performed in triplicate. Representative immunoblots, plates and mean colony numbers ± standard errors are shown. (E) After the lentiviral modification of RB1CC1 expression, HeLa cells were analyzed by flow cytometry. RB1CC1 and sh-RB1CC1 are indicated by red (left) and blue (right), respectively. The data (mean percentages ± standard deviations) were confirmed in triplicate experiments, and a representative flow cytometric graph is demonstrated. Arrows indicate whether statistically significant increases (up-arrow) or decreases (down-arrow) are present according to the Student's t-test; p<0.05.
(0.68 MB TIF)

Table S1 The primer sequences and conditions for ChIP-PCR and RT-PCR.
(0.04 MB DOC)

## References

[1] Giacinti C, Giordano A (2006). RB and cell cycle progression.. Oncogene.

[2] Chano T, Ikegawa S, Kontani K, Okabe H, Baldini N (2002). Identification of RB1CC1, a novel human gene that can induce RB1 in various human cells.. Oncogene.

[3] Chano T, Kontani K, Teramoto K, Okabe H, Ikegawa S (2002). Truncating mutations of RB1CC1 in human breast cancer.. Nat Genet.

[4] Ikebuchi K, Chano T, Ochi Y, Tameno H, Shimada T (2009). RB1CC1 activates the promoter and expression of RB1 in human cancer.. Int J Cancer.

[5] Schmidt-Kittler O, Ragg T, Daskalakis A, Granzow M, Ahr A (2003). From latent disseminated cells to overt metastasis: genetic analysis of systemic breast cancer progression.. Proc Natl Acad Sci U S A.

[6] Melkoumian ZK, Peng X, Gan B, Wu X, Guan JL (2005). Mechanism of cell cycle regulation by FIP200 in human breast cancer cells.. Cancer Res.

[7] Betz BL, Strobeck MW, Reisman DN, Knudsen ES, Weissman BE (2002). Re-expression of hSNF5/INI1/BAF47 in pediatric tumor cells leads to G1 arrest associated with induction of p16ink4a and activation of RB.. Oncogene.

[8] Lee D, Kim JW, Seo T, Hwang SG, Choi EJ (2002). SWI/SNF complex interacts with tumor suppressor p53 and is necessary for the activation of p53-mediated transcription.. J Biol Chem.

[9] Chai J, Charboneau AL, Betz BL, Weissman BE (2005). Loss of the hSNF5 gene concomitantly inactivates p21CIP/WAF1 and p16INK4a activity associated with replicative senescence in A204 rhabdoid tumor cells.. Cancer Res.

[10] Gresh L, Bourachot B, Reimann A, Guigas B, Fiette L (2005). The SWI/SNF chromatin-remodeling complex subunit SNF5 is essential for hepatocyte differentiation.. Embo J.

[11] Sakai T, Ohtani N, McGee TL, Robbins PD, Dryja TP (1991). Oncogenic germ-line mutations in Sp1 and ATF sites in the human retinoblastoma gene.. Nature.

[12] Martin N, Schwamborn K, Urlaub H, Gan B, Guan JL (2008). Spatial interplay between PIASy and FIP200 in the regulation of signal transduction and transcriptional activity.. Mol Cell Biol.

[13] Hara T, Takamura A, Kishi C, Iemura S, Natsume T (2008). FIP200, a ULK-interacting protein, is required for autophagosome formation in mammalian cells.. J Cell Biol.

[14] Hosokawa N, Hara T, Kaizuka T, Kishi C, Takamura A (2009). Nutrient-dependent mTORC1 Association with the ULK1-Atg13-FIP200 Complex Required for Autophagy.. Mol Biol Cell.

[15] Hara T, Mizushima N (2009). Role of ULK-FIP200 complex in mammalian autophagy: FIP200, a counterpart of yeast Atg17?. Autophagy.

[16] Young AR, Narita M, Ferreira M, Kirschner K, Sadaie M (2009). Autophagy mediates the mitotic senescence transition.. Genes Dev.

[17] Chano T, Saji M, Inoue H, Minami K, Kobayashi T (2006). Neuromuscular abundance of RB1CC1 contributes to the non-proliferating enlarged cell phenotype through both RB1 maintenance and TSC1 degradation.. Int J Mol Med.

[18] Sarbassov DD, Guertin DA, Ali SM, Sabatini DM (2005). Phosphorylation and regulation of Akt/PKB by the rictor-mTOR complex.. Science.

[19] Yagi A, Hasegawa Y, Xiao H, Haneda M, Kojima E (2003). GADD34 induces p53 phosphorylation and p21/WAF1 transcription.. J Cell Biochem.

