# Supplementary figures and images for: RB1CC1 Activates RB1 Pathway and Inhibits Proliferation and Cologenic Survival in Human Cancer
Source: PLoS One. 2010 Jun 30;5(6):e11404. doi: 10.1371/journal.pone.0011404 (PMC2894861; doi:10.1371/journal.pone.0011404)

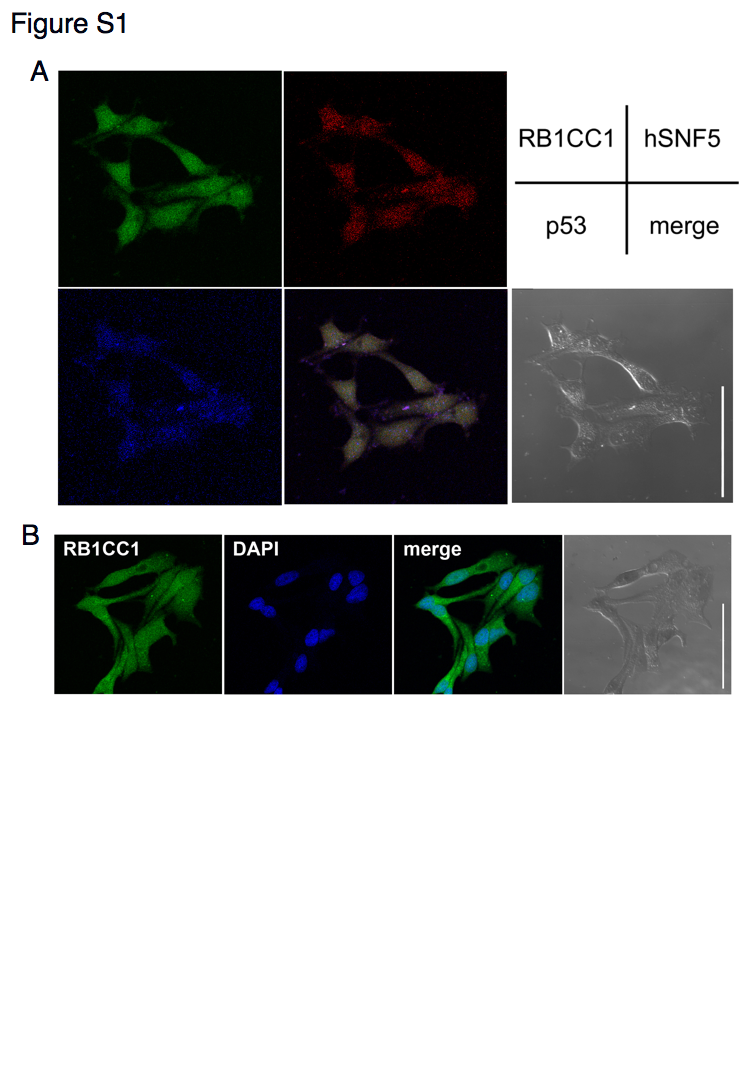

Supplement: Figure S1 — RB1CC1 interacts with hSNF5 and p53 mainly in cell nuclei. (A) RB1CC1 (green), hSNF5 (red), p53 (blue) and the merged image in MCF-7 cells. Each protein is immunofluorescently labeled by Alexa 488, 594 or 350 respectively. Scale bar, 50µm. (B) RB1CC1 (green), DAPI and the merged image are evaluated in MCF-7 cells. RB1CC1 is immuno-labeled by Alexa 488, and DAPI is displayed with blue fluorescence in cell nuclei. Scale bar, 50µm. (0.90 MB TIF) [file pone.0011404.s001.tif]

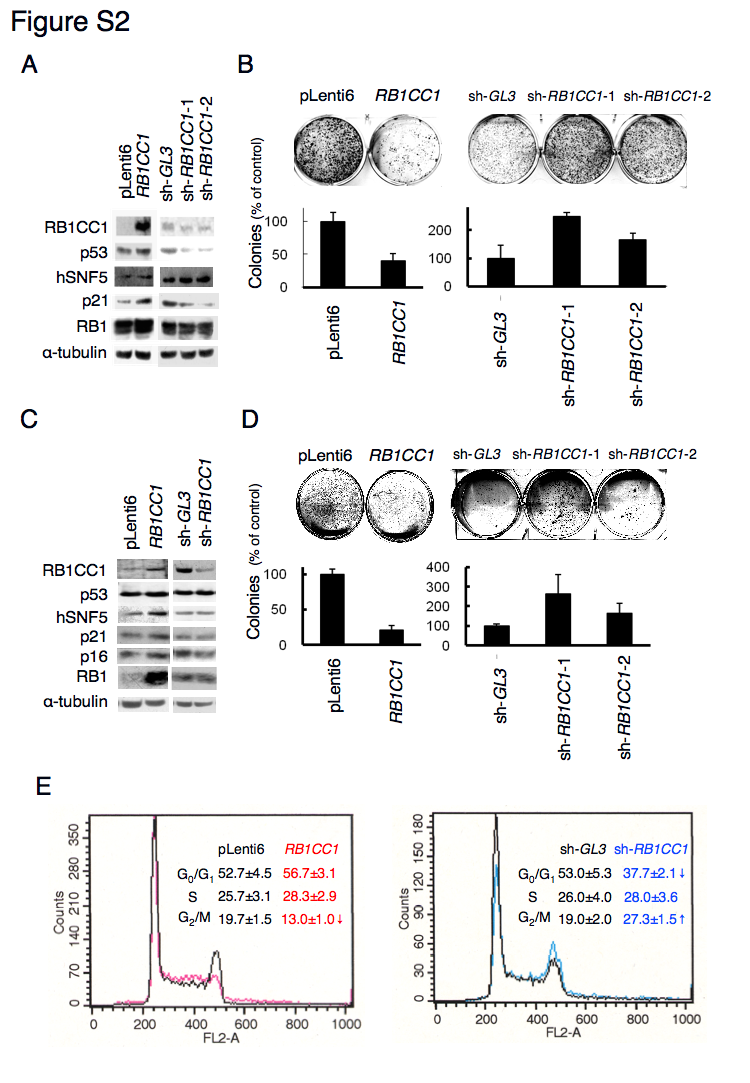

Supplement: Figure S2 — Modulation of RB1CC1 affects the growth of breast cancer cells through RB1, p21 and/or p16 expression. (A) In MCF-7 breast cancer cells, RB1CC1 induced RB1, p53 and p21 expression, and, conversely, the knockdown (sh-RB1CC1) reduced their expression. (B) RB1CC1 suppressed and sh-RB1CC1 enhanced the cell growth of MCF-7. (C) In SK-BR3 breast cancer cells, RB1CC1 induced RB1, hSNF5, p16 and p21 expression. sh-RB1CC1 decreased the p16 and p21 contents. (D) RB1CC1 suppressed and sh-RB1CC1 enhanced the cell growth of SK-BR3. A and C indicate the Western blots data. B and D show the results of cell growth assays. Modulation was performed by lentiviral transduction of cDNA or sh-RNA of RB1CC1 into the cells. pLenti6 and sh-GL3 were used as controls. The experiments were performed in triplicate. Representative immunoblots, plates and mean colony numbers ± standard errors are shown. (E) After the lentiviral modification of RB1CC1 expression, HeLa cells were analyzed by flow cytometry. RB1CC1 and sh-RB1CC1 are indicated by red (left) and blue (right), respectively. The data (mean percentages ± standard deviations) were confirmed in triplicate experiments, and a representative flow cytometric graph is demonstrated. Arrows indicate whether statistically significant increases (up-arrow) or decreases (down-arrow) are present according to the Student's t-test; p<0.05. (0.68 MB TIF) [file pone.0011404.s002.tif]
