# Supplementary material for: RB1CC1 Activates RB1 Pathway and Inhibits Proliferation and Cologenic Survival in Human Cancer
Source: PLoS One. 2010 Jun 30;5(6):e11404. doi: 10.1371/journal.pone.0011404 (PMC2894861; doi:10.1371/journal.pone.0011404)
Supplement: Table S1 — The primer sequences and conditions for ChIP-PCR and RT-PCR. (0.04 MB DOC) [file pone.0011404.s003.doc]

**Table S1.** The primer sequences and conditions for ChIP-PCR and RT-PCR

| Gene  (Accession No.) | Primer Sequence  (5’ to 3’) | Product Size (bp) | Tm  (°C) |
| --- | --- | --- | --- |
| *RB1* promoter  (NT_024524) | F: GGGGTGGTTCTGGGTAGAAG  R: AATTTCAACGTCCCCTGAGA | 275 | 60 * |
| *p16* promoter  (NT_007592) | F: AGGAATCCTTTGAACTAGGG  R: GGGAAAGTATGGCTTCTTCT | 183 | 58 |
| *p21* promoter  (NT_008413) | F: CACCTTTCACCATTCCCCTA  R: CCCTTCCTCACCTGAAAACA | 257 | 62 |
| *RB1CC1*  (NM_014781) | F: CCAGGAGCAAGACAGAGAAC  R: CGAGCTCGATCTTCAGAAAG | 465 | 56 |
| *hSNF5*  (NM_003073 ) | F: TGCATCGTCACATGGTAAAAA  R: CTCAGGCGTCATCAACTTCTC | 457 | 56 |
| *p53*  (NM_000546) | F: GCGCACAGAGGAAGAGAATC  R: TGAGTCAGGCCCTTCTGTCT | 331 | 56 |
| *p21*  (NM_078467) | F: GCGACTGTGATGCGCTAATG  R: AGAAGATCAGCCGGCGTTTG | 384 | 55 |
| *p16*  (NM_000077) | F: CTTCCTGGACACGCTGGT  R: GGATGTCTGAGGGACCTTCC | 197 | 55 |
| *RB1*  (NM_000321) | F: ACCAGATCATGTCAGAGAGA  R: ACTGCTGGGTTGTGTCAAAT | 340 | 55 |
| *GAPDH*  (NM_002046) | F: AACTTTGGTATCGTGGAAGGACT  R: CTGTAGCCAAATTCGTTGTCATAC | 464 | 55 |

The PCR conditions was as follows with the Takara Ex Taq^TM^ DNA polymerase (TaKaRa): One cycle at 95°C for 3 min, and followed by multiple cycles at 95°C for 20 sec, Tm °C for 20 sec, 72°C for 30 sec.

*: For ChIP-PCR of *RB1* promoter, the PCR was composed of multiple cycles at 98°C for 10 sec, 60°C for 20 sec, 72°C for 30 sec, using the Pyrobest^TM^ DNA polymerase (TaKaRa).
